# Effect of Nadir CD4+ T Cell Count on Clinical Measures of Periodontal Disease in HIV+ Adults before and during Immune Reconstitution on HAART 

**DOI:** 10.1371/journal.pone.0076986

**Published:** 2013-10-11

**Authors:** Lance T. Vernon, Catherine A. Demko, Denise C. Babineau, Xuelei Wang, Zahra Toossi, Aaron Weinberg, Benigno Rodriguez

**Affiliations:** 1 Department of Biological Sciences, Case Western Reserve University School of Dental Medicine, Cleveland, Ohio, United States of America; 2 Department of Community Dentistry, Case Western Reserve University School of Dental Medicine, Cleveland, Ohio, United States of America; 3 Center for Clinical Investigation, Case Western Reserve University, Cleveland, Ohio, United States of America; 4 Department of Medicine, Division of Infectious Diseases, Case Western Reserve University, Cleveland, Ohio, United States of America; 5 Center for AIDS Research, Case Western Reserve University/University Hospitals of Cleveland, Cleveland, Ohio, United States of America; New York University, United States of America

## Abstract

**Background:**

The contribution of HIV-infection to periodontal disease (PD) is poorly understood.  We proposed that immunological markers would be associated with improved clinical measures of PD.

**Methods:**

We performed a longitudinal cohort study of HIV-infected adults who had started highly active antiretroviral therapy (HAART) <2 years. PD was characterized clinically as the percent of teeth with ≥1 site with periodontal probing depth (PPD) ≥5.0mm, recession (REC) >0mm, clinical attachment level (CAL) ≥4.0mm, and bleeding on probing (BOP) at ≥4 sites/tooth and microbiologically as specific periodontopathogen concentration. Linear mixed-effects models were used to assess the associations between immune function and PD.

**Results:**

Forty (40) subjects with median 2.7 months on HAART and median nadir CD4+ T-cell count of 212 cells/μl completed a median 3 visits. Over 24 months, CD4+ T-cell count increased by a mean 173 cells/µl (p<0.001) and HIV RNA decreased by 0.5 log_10_ copies/ml (p<0.001); concurrently, PPD, CAL and BOP decreased by a mean 11.7%, 12.1%, and 14.7% respectively (all p<0.001). Lower nadir CD4+ T-cell count was associated with worse baseline REC (-6.72%; p=0.04) and CAL (9.06%; p<0.001). Further, lower nadir CD4+ T-cell count was associated with a greater relative longitudinal improvement in PPD in subjects with higher baseline levels of *Porphyromonas gingivalis* (p=0.027), and BOP in subjects with higher baseline levels of *Porphyromonas gingivalis* or *Treponema denticola* (p=0.001 and p=0.006 respectively). Longitudinal changes from baseline in CD4+ T-cell count and level of HIV RNA were not independently associated with longitudinal changes in any clinical markers of PD.

**Conclusion:**

Degree of immunosuppression was associated with baseline gingival recession. After HAART initiation, measures of active PD improved most in those with lower nadir CD4+ T-cell counts and higher baseline levels of specific periodontopathogens. Nadir CD4+ T-cell count differentially influences periodontal disease both before and after HAART in HIV-infected adults.

## Background

While HIV/AIDS is considered a “modifier” of periodontal disease (PD) [1,2], the mechanisms of this connection are poorly understood. Clearly, immunosuppression can potentiate PD, as evidenced by reports of florid examples of HIV-associated gingival/periodontal disease during the early days of HIV outbreak in the United States [3,4]. Recently, in a cross-sectional evaluation of 112 HIV+ adults, our group found that ever having a CD4+ T-cell count below 200 cells/μl conferred approximately twice the risk for traditionally defined PD as did cigarette smoking, a known strong risk factor for PD [5]. Even in the era of highly active antiretroviral therapy (HAART), the prevalence of traditionally defined PD within cohorts of predominantly African American HIV+ adult cohorts has been high, ranging from 66% to >90% [5,6] depending on the definition of PD used. Because African Americans are disproportionately infected with HIV[7], the public health relevance of traditionally-defined PD in HIV+ adults is significant both in terms of the population affected and patient morbidity; however, the issue remains underappreciated and under-recognized. There are several methodological limitations in earlier studies proposing a low level of PD in HIV+ adult cohorts, as detailed in our 2009 report[5]. In our 2011 longitudinal report of 43 HIV+ patients on HAART, we found that PD improved significantly during immune reconstitution on HAART[8]. We subsequently hypothesized that longitudinal improvement in CD4+ T-cell count and/or decreased level of HIV RNA would be associated with longitudinal improvement in clinical measures of PD. To address this hypothesis, we extended the analysis of our earlier report[8] by modeling the effect of nadir CD4+ T cell count and the longitudinal change in CD4+ T cell count and level of HIV RNA on clinical measures of PD. We found that nadir CD4+ T cell count influences periodontal disease in HIV-infected adults both before and after HAART initiation, and that this influence varies prior to and after HAART initiation. 

## Methods

### Study Design

This was a prospective observational study of adult subjects recruited from three outpatient HIV medical clinics in Cleveland, Ohio as previously described[5,8]. IRB approval was obtained from University Hospitals Case Medical Center (UHCMC). Most participants were self-referred; all subjects signed a written UHCMC IRB-approved informed consent document. Exclusion criteria included evidence of cardiovascular disease, a history of Type I or II diabetes mellitus, fewer than 20 teeth, uncontrolled systemic illnesses, diagnosis or treatment of cancer in the past five years, pregnancy, and need for antibiotic prophylaxis prior to dental care as per the American Dental Association (ADA) and other guidelines[9,10]. Inclusion criteria were: medication-compliant adult subjects, age 18 or older, who were taking highly active antiretroviral therapy (HAART) for < 2 years at baseline. HAART was defined previously[8] as a treatment regimen that included at least three different antiretroviral drugs from at least two different classes. All subjects were seen for evaluation and PD measurement at baseline and at one or more visits thereafter, typically at 8, 16 and 24 months. Study subjects were seen from May, 2005 through January, 2008. 

### Periodontal Disease Measurements and Definition

The periodontal probing depth (PPD), gingival recession (REC), clinical attachment level (CAL) and bleeding on probing (BOP) were determined at six sites per tooth by one dentist (LTV) as previously described[5]. The percent agreement from ongoing intra-rater reliability for periodontal probing (+/- 1 mm) was 98% with an intra-class correlation coefficient of 0.88. A viable tooth was defined as having at least one-half of a remaining clinical crown and having at least three contiguous sites in which PPD, REC and CAL were measurable. To ensure probing accuracy, supragingival plaque, debris, blood and saliva were removed prior to performing precise full-mouth periodontal measurements. To minimize the possibility of misclassification by categorizing data, we defined and analyzed PD data as continuous variables. Herein, we report PD as per its component parts, PPD, REC and the summary measure, CAL[5,11,12], because these components may represent different processes that contribute to periodontal disease[11,12], and each component outcome[11,12] may help illuminate how HIV-1 infection influences or interacts with the progression of PD[5]. We define BOP as the percent of teeth with BOP on ≥4 sites per tooth. Consistent with our previous report, we used the following thresholds to define periodontal disease: PPD >5 mm, REC >0 mm and CAL ≥4mm; thus, for each component, we calculated the percent of teeth with at least one site per tooth that met the threshold and analyzed each outcome as a continuous variable. 

### Study Measures

Clinical data were obtained from the primary HIV clinic’s longitudinal electronic database or from retrospective chart reviews. One dentist researcher (LTV) administered questionnaires to obtain demographic, medical and oral behavioral data as previously reported[5]. Smoking was recorded as ever having smoked more than 100 cigarettes and pack-per-day years (ppdyrs) of cigarette smoking. 

### Clinical Measures

CD4+ T cell count was measured by flow cytometry and HIV RNA by polymerase chain reaction (PCR) as previously described[5,8] Data on CD4+ T-cell count and HIV RNA was merged with periodontal measures using a window on immunological variables defined by less than 4 months before or 1 week after each study visit.

### Collection of Dental Plaque for Periodontal Pathogens

To collect subgingival dental plaque, a separate sterile 13/14 Gracey curette was used for each tooth. Plaque was harvested from the most apical portion of the probing depth alongside the surface of the tooth’s root at two different interproximal sites (usually the distobuccal and distolingual) of the first molar in each quadrant[5]. For each patient, plaque was obtained from eight molar tooth sites and transferred into a single microcentrifuge tube that contained 0.5 ml tri-reagent (Molecular Research Center Inc., Cincinnati, Ohio). If the first molar tooth in a given quadrant was missing, the next most distal tooth was accessed. If all molar teeth were missing, dental plaque from the most distal tooth in that quadrant was harvested, as described previously[13]. After placing on ice temporarily, the microcentrifuge tubes were stored in a -70°C freezer until DNA extraction was performed. 

### Isolation and quantification of DNA

DNA from dental plaque samples was extracted according to the manufacturer’s instructions as previously described[14]. Bacterial DNA for *Porphyromonas gingivalis* (*Pg*), *Treponema denticola* (*Td*) and *Tanneralla forsythia* (*Tf*)*,*as well as DNA encoding total bacterial 23s ribosomal RNA was assessed using real-time PCR (Taqman 7700, Applied Biosystems, Foster City, CA). Primer Express software (Applied Biosystems) was used to design Taqman primer and probes for *Pg, Td*, and bacterial 23s ribosomal RNA, while primers and probe for *Tf* were replicated as per Morillo et al., 2004[15]. For details on probes and primers, see our previous reports[5,8] (NCBI accession numbers D64081.1, U29399.1and AY423857.1, for *Pg, Td* and *Tf*, respectively). Products of bacterial 23 S ribosomal DNA were detected using SYBR Green (Applied Biosystems). Bacterial DNA quantities were determined using a dilution series of oligonucleotide sequences (Invitrogen, Carlsbad, CA) synthesized for each assay’s target DNA. Plaque DNA concentration and purity were determined using spectrophotometery (BECKMAN DU640). After correcting for the total DNA in each plaque sample, DNA copies for each pathogen were expressed as copies of bacterial DNA per microgram of DNA. 

### Statistical Analysis

Baseline variables were summarized using standard descriptive statistics. To characterize disease progression during the study, the average change in immunological markers as well as clinical periodontal disease markers over a 24 month period was estimated using separate linear mixed-effects models that included a random intercept and slope with a compound symmetry correlation structure to account for between-subject variance. The association between nadir CD4+ T-cell count and baseline clinical periodontal disease markers was estimated using separate linear regression models adjusting for age and total pack-per-day years of cigarette smoking. Because CD4+ T-cell count and HIV RNA levels changed throughout the study, separate linear mixed effects models were also used to simultaneously estimate the cross-sectional (or between-subject) association between baseline CD4+ T-cell count (or HIV RNA) and baseline clinical PD measures, as well as the longitudinal (or within-subject) association between changes in CD4+ T-cell count (or HIV RNA) over time and changes in clinical PD measures over time. Models included fixed effects for time on study, baseline CD4+ T-cell count (or HIV RNA), change in CD4+ T-cell count (or HIV RNA) from baseline, age, and total pack-per-day years of cigarette smoking. Linear mixed models were also used to estimate the association between nadir CD4+ T-cell count and the rate of change in clinical measures of periodontal disease over time. Models included fixed effects for time on study, nadir CD4+ T-cell count, age, and total pack-per-day years of cigarette smoking as well as associated pairwise interactions with time on study. To investigate whether this effect was modified by baseline periodontal pathogens, a 3-way interaction between time on study, nadir CD4+ T-cell count and baseline periodontal pathogens was also included in all models. Data were analyzed using SAS (Version 9.2; Cary, NC).

## Results and Discussion

### Results

There were 50 patients in our study who had at least one follow-up periodontal visit. Of these, 10 patients were excluded because they were not on HAART at the baseline visit (n=6), not on HAART during the study (n=2), had a full mouth extraction before any follow-up visits (n=1) and because CD4+ T-cell count was not available 4 months before or 1 week after the baseline visit (n=1). Among the 40 patients in the final analyses, 8 (20%) had one follow-up periodontal (examination) visit, 13 (32.5%) had two follow-up periodontal visits, 12 (30%) had 3 follow-up periodontal visits, 3 (7.5%) had 4 follow-up periodontal visits and 4 (10%) had 5 follow-up periodontal visits, for a total of 142 study visits. The Spearman correlation between baseline CD4+ and nadir CD4+ T-cell count was 0.81 (p<0.01). The Spearman correlation between baseline CD4+ T-cell count and baseline plasma HIV RNA was -0.33 (p=0.04). The correlation between nadir CD4+ T-cell count and baseline HIV RNA was weak with a Spearman correlation of 0.04. Across the study, the median number of teeth extracted was 0 (range 0-10); a total of 40 teeth were extracted from 11 subjects (27.5%). At end of study, 28 (70%) had achieved a viral load below the limit of detection of commercial assays (HIV RNA ≤50 copies/mL). About half of all subjects (53.5%) were seen by a dentist prior to their follow-up study visit, but only 7 subjects (17.5%) received scaling and root planing (SC/RP) throughout the study (i.e., prior to 10.8% of all recall visits). 

Table 1 summarizes baseline characteristics for all 40 subjects included in the analytic sample. At baseline, subject’s mean age was 37.4 ± 8.6 years. Most subjects (70%) were male and a majority of participants were black (59%). Overall, subjects had recently started HAART; the median time on HAART at baseline was 2.7 (IQR: 1.1-8.3) months and the median nadir CD4+ T-cell count was 211.5 (inter-quartile range (IQR): 116.5-295.0) cells/μl. Total mean study follow-up time was 22.8 ±10.3 months. Each subject had a median of 3 (range 2-6) study visits during which the extent of periodontal disease was measured. On average, subjects were on HAART during 97% of study visits. The median baseline CD4+ T-cell count was 346.0 (IQR: 203.0-454.0) cells/µl and the median HIV RNA was 400.0 (IQR: 53.5-15530) copies/ml. For additional patient characteristics during the follow-up period, see Table S1.

**Table 1:** Summary statistics for patients at baseline (N=40).

**Baseline characteristics**	**Mean (SD)**
	**Frequency (Proportion)**
**Demographics**	
Age (years)	37.4(8.6)
Male	28(70.0%)
Black	23(59.0%)
BMI (kg/m^2^)	26.3(7.5)
Ever smoked	29(72.5%)
Total pack-per-day years smoked	8.0(11.3)
≤ High school graduate/GED	31(77.5%)
Medicaid/care or Ryan White insurance	26(65.0%)
**HIV specific**	
Months since first seropositive test	44.5(54.9)
Total months on HAART	5.7(6.0)
Months between nadir CD4+ T-cell count and baseline CD4+ T-cell count	8.7(13.4)
**Immune/Inflammatory variables**	
CD+ T-cell count (cells/µl)	348.5(191.9)
Nadir CD4+ T-cell count (cells/µl)	210.1(140.3)
HIV RNA viral load (copies/mL)	32643(69158)
HIV RNA “undetectable” (≤50 copies/mL)	10 (25%)
**Clinical periodontal disease measures**	
PPD ≥5.0 mm (% teeth)	33.5(23.4)
REC >0 mm (% teeth)	47.1(31.8)
CAL ≥4 mm (% teeth)	46.5(32.4)
BOP on ≥4 sites/tooth (% teeth)	40.9(24.7)

GED, general educational development; BMI, Body Mass Index; PPD, periodontal probing depth; REC, gingival

recession; CAL, clinical attachment level; BOP, bleeding on probing.

### Change in immunological and virological markers and clinical measures of PD over time

Table 2 describes estimated disease progression over a 24-month period. On average, our cohort experienced immune reconstitution during the study; CD4+ T-cell count increased by 173.1 cells/µl (95% CI: (105.3, 240.9); p<0.001) and HIV RNA decreased by 0.5 log_10_ copies/ml (95% CI: (0.3, 0.8); p<0.001). Additionally, on average, PPD, CAL and BOP decreased by 11.7% (95% CI: (7.6%, 15.8%); p<0.001), 12.1% (95% CI: (7.9%, 16.4%); p<0.001) and 14.7% (95% CI: (8.7%, 20.7%); p<0.001) respectively. 

**Table 2:** Estimated mean at baseline and mean change between baseline and 24 months for characteristics of interest.

**Characteristic**	**Mean (SD) at baseline**	**Estimated mean change (95% CI) between baseline and 24 months**	**p-value**
**Demographics**			
BMI (kg/m^2^)	26.3(7.5)	1.4 (0.5, 2.2)	0.003
**Immune/Inflammatory variables**			
CD4+ T-cell count (cells/µl)	348.5(191.9)	173.1 (105.3, 240.9)	< 0.001
HIV RNA (log_10_ copies/µl)	3.0(1.3)	-0.5 (-0.8, -0.3)	< 0.001
**Clinical periodontal disease measures**			
PPD ≥5.0 mm (% teeth)	33.5(23.4)	-11.7 (-15.8, -7.6)	< 0.001
REC >0 mm (% teeth)	47.1(31.8)	-4.5 (-9.5, 0.5)	0.08
CAL ≥4 mm (% teeth)	46.5(32.4)	-12.1 (-16.4, -7.9)	< 0.001
BOP on ≥4 sites/tooth (% teeth)	40.9(24.7)	-14.7 (-20.7, -8.7)	< 0.001

BMI, Body Mass Index; PPD, periodontal probing depth; REC, gingival recession; CAL, clinical attachment level; BOP, bleeding on probing.

### Associations between baseline immunological and virological markers and baseline measures of PD

A summary of the cross-sectional association between nadir CD4+ T-cell count and baseline measures of PD is provided in Table 3. After controlling for age and smoking pack-per-day years, lower nadir CD4+ T-cell count was associated with worse (higher) baseline REC (6.72% per 100 cells/µl decrease in CD4+ T-cell count; 95% CI: (0.43%, 13.01%); p=0.04) and CAL (9.06% per 100 cells/µl decrease in CD4+ T-cell count; 95% CI: (4.34%, 13.79%); p<0.001), but not PPD or BOP. There was no evidence of an association between nadir CD+ T-cell count and the baseline levels of any periodontal pathogens (p≥0.06; see Table 3). Similarly, in adjusted models that simultaneously estimated the cross-sectional and longitudinal associations between CD4+ T-cell count and clinical PD measures, lower baseline CD4+ T-cell count was also associated with a higher baseline REC (4.18% increase per 100 cells/µl decrease in CD4+ T-cell count; 95 CI: (0.07%, 8.43%); p=0.05) and CAL (6.10% increase per 100 cells/µl decrease in CD4+ T-cell count; 95% CI: (1.85%, 10.35%); p=0.01) but not with PPD or BOP (p≥0.14; see Table 4). There was no evidence of an association between baseline HIV RNA and any of the baseline clinical measures of PD (p≥0.23; see Table 4). 

**Table 3:** Cross-sectional association between nadir CD4+ T cell count and baseline clinical and microbial measures of PD.

		***Increase in baseline clinical PD measure per 100 cells/µl increase in nadir CD4+ T cell count***			
**Clinical measure of PD**	Model^*^	***Mean estimate (95%CI)***		***p-value***	
PPD	1	-1.57 (-5.81,2.67)		0.46	
PPD	2	-1.70 (-6.01,2.61)		0.43	
REC	1	-6.29 (-14.29,1.70)		0.12	
REC	2	-6.72 (-13.01,-0.43)		0.04	
CAL	1	-8.72 (-14.30,-3.13)		0.003	
CAL	2	-9.06 (-13.79,-4.34)		<0.001	
BOP	1	-2.74 (-8.61,3.12)		0.35	
BOP	2	-2.57 (-8.52,3.38)		0.39	
		***Increase in baseline microbial PD measure per 100 cells/µl increase in nadir CD4+ T cell count***		
**Microbial measure of PD**	Model^*^	***Mean estimate (95%CI)***	***p-value***	
*Pg*	1	-0.14 (-0.65,0.37)	0.57	
*Pg*	2	-0.15 (-0.64,0.34)	0.54	
23s rDNA	1	-0.09 (-0.19,0.02)	0.10	
23s rDNA	2	-0.09 (-0.19,0.02)	0.09	
*Td*	1	0.15 (-0.16,0.45)	0.34	
*Td*	2	0.15 (-0.18,0.47)	0.37	
*Tf*	1	0.22 (-0.01,0.46)	0.07	
*Tf*	2	0.23 (-0.01,0.47)	0.06	

* **Model 1**: Baseline Nadir CD4+ T-cell count; **Model 2:** Model 1 + age + total pack-per-day years of

cigarette smoking. PD, periodontal disease; PPD, periodontal probing depth, REC, gingival recession;

CAL, clinical attachment level; BOP, bleeding on probing; *Pg, Porphyromonas gingivalis;* 23s rDNA,

Total 23s ribosomal DNA; *Td, Treponema denticola; Tf, Tanneralla forsythia.*

**Table 4:** Cross-sectional and longitudinal associations between CD4+ T-cell count and *HIV RNA with clinical measures of periodontal disease* .

		**Increase in clinical PD per 100 cells/µl increase in baseline CD4+ T-cell count**		**Increase in the change in clinical measure of PD from baseline per 100 cells/µl increase in the change in CD4+ T-cell count from baseline**	
**Clinical PD measure**	Model^*^	***Mean estimate (95%CI)***	***p-value***	***Mean estimate (95%CI)***	***p-value***
PPD	1	-1.82 (-4.58,0.93)	0.19	0.90 (-1.46,3.26)	0.45
PPD	2	-2.02 (-4.75,0.71)	0.14	0.91 (-1.49,3.32)	0.45
REC	1	-3.43 (-8.79,1.94)	0.21	-0.03 (-1.45,1.39)	0.97
REC	2	-4.18 (-8.43,0.07)	0.05	-0.05 (-1.43,1.34)	0.94
CAL	1	-5.66 (-10.74,-0.59)	0.03	-0.24 (-1.28,0.79)	0.64
CAL	2	-6.10 (-10.35,-1.85)	0.01	-0.28 (-1.33,0.76)	0.59
BOP	1	-1.28 (-4.33,1.77)	0.41	1.88 (-0.46,4.21)	0.11
BOP	2	-1.10 (-4.27,2.08)	0.49	1.87 (-0.50,4.25)	0.12
		**Increase in baseline clinical PD measure per 1-log_10_ increase in baseline HIV RNA**		**Increase in the change in clinical measure of PD from baseline per 1-log_10_ increase in the change in HIV RNA from baseline**	
**Clinical PD measure**	Model^*^	***Mean estimate (95%CI)***	***p-value***	***Mean estimate (95%CI)***	***p-value***
PPD	1	-2.93 (-6.98,1.12)	0.15	-0.32 (-2.36,1.71)	0.75
PPD	2	-2.37 (-6.31,1.56)	0.23	-0.29 (-2.34,1.76)	0.78
REC	1	-1.41 (-8.45,5.63)	0.69	0.40 (-1.68,2.47)	0.70
REC	2	3.07 (-2.82,8.96)	0.30	0.50 (-1.60,2.59)	0.64
CAL	1	-1.27 (-8.56,6.02)	0.73	0.08 (-1.86,2.02)	0.94
CAL	2	2.19 (-4.27,8.66)	0.50	0.15 (-1.81,2.12)	0.88
BOP	1	-0.76 (-5.68,4.16)	0.76	0.91 (-1.82,3.64)	0.51
BOP	2	-2.06 (-7.49,3.37)	0.45	0.83 (-1.85,3.52)	0.54

* **Model 1**: Time on study + baseline CD4+ T-cell count + change in CD4+ T-cell count from baseline; **Model 2**: Model 1 + age + total pack-per day years of cigarette smoking.

### Longitudinal effect of nadir CD4+ T-cell count on rate of change in clinical measures of PD

As shown in Table 5, lower nadir CD4+ T-cell count was associated with a greater relative longitudinal improvement (i.e., decrease) in: 1) PPD in subjects with higher baseline levels of *Pg* than in subjects with lower baseline levels of *Pg* (p=0.027). Similarly, lower nadir CD4+ T-cell count was associated with a greater decrease over time in: 1) BOP in subjects with higher baseline levels of *Pg* or *Tf*, than in subjects with lower levels of baseline *Pg* or *Tf* (p=0.001 and p=0.006 respectively); and in 2) REC in subjects with higher baseline levels of 23s rDNA than in subjects with lower levels of 23s rDNA (p=0.031). Finally, there was no evidence of a longitudinal association between nadir CD4+ T-cell count and the rate of change in CAL (p-value=0.12). 

**Table 5:** Association between nadir CD4+ T cell count and the longitudinal rate of change in clinical periodontal measures adjusting for baseline level of periodontal pathogens.

		**Mean difference in rate of change in PPD per 12 months**		**Mean difference in rate of change in REC per 12 months**	
**Model**	Exposure*	***Mean estimate (95%CI)***	***p-value***	***Mean estimate (95%CI)***	***p-value***
1	Nadir CD4	-0.04 (-1.39,1.32)	0.959	1.84 (0.23,3.45)	0.026
2	Nadir CD4	0.00 (-1.45,1.46)	0.996	1.89 (0.20,3.58)	0.029
3	Nadir CD4	**0.01 (-1.47,1.50**)	**0.984^a^**	1.83 (0.54,3.11)	0.006
3	*Pg*	**-0.58 (-1.68,0.51**)	**0.292^a^**	-1.51 (-2.61,-0.42)	0.008
4	Nadir CD4	-0.10 (-1.50,1.30)	0.885	**1.89 (0.01,3.77**)	**0.049^b^**
4	23s rDNA	-1.55 (-6.12,3.02)	0.500	**-0.20 (-6.93,6.54**)	**0.954^b^**
5	Nadir CD4	0.26 (-1.28,1.81)	0.733	1.97 (0.27,3.68)	0.024
5	*Td*	-0.92 (-2.24,0.39)	0.164	-0.27 (-1.55,1.01)	0.670
6	Nadir CD4	0.23 (-1.33,1.79)	0.766	1.89 (0.27,3.51)	0.023
6	*Tf*	-0.80 (-2.72,1.12)	0.409	0.06 (-1.18,1.30)	0.921
		**Mean difference in rate of change in CAL per 12 months**		**Mean difference in rate of change in BOP per 12 months**	
**Model**	Exposure*	***Mean estimate (95%CI)***	***p-value***	***Mean estimate (95%CI)***	***p-value***
1	Nadir CD4	1.08 (-0.11,2.27)	0.075	0.12 (-1.92,2.16)	0.910
2	Nadir CD4	1.04 (-0.27,2.36)	0.117	**0.06 (-1.96,2.08**)	0.953
3	Nadir CD4	1.00 (-0.24,2.24)	0.113	**0.08 (-2.12,2.29**)	**0.939^a^**
3	*Pg*	-0.83 (-2.22,0.56)	0.237	**-1.67 (-3.17,-0.16**)	**0.030^a^**
4	Nadir CD4	1.13 (-0.13,2.39)	0.078	**-0.13 (-2.00,1.73**)	0.888
4	23s rDNA	1.16 (-3.24,5.55)	0.600	**-1.60 (-8.68,5.47**)	0.652
5	Nadir CD4	1.03 (-0.41,2.46)	0.158	**0.55 (-1.60,2.70**)	0.610
5	*Td*	0.08 (-1.22,1.39)	0.899	**-1.66 (-3.29,-0.02**)	0.048
6	Nadir CD4	0.90 (-0.44,2.24)	0.182	**0.70 (-1.39,2.79**)	**0.506^c^**
6	*Tf*	0.52 (-0.69,1.73)	0.394	**-1.95 (-4.01,0.12**)	**0.064^c^**

**Model 1:** Unadjusted.

**Models 2, 3, 4, 5 and 6**: Adjusted for age and total pack-per-day years of cigarette smoking at baseline.

Nadir CD4, nadir CD4+ T cell count; PPD, periodontal probing depth, REC, gingival recession; CAL, clinical attachment level; BOP, bleeding on probing; *Pg*, *Porphyromonas* *gingivalis*; 23s rDNA, total 23s ribosomal DNA; *Td*, *Treponema* *denticola*; *Tf*, *Tanneralla* *forsythia.*

a Interaction between baseline nadir CD4+ and log_10_ *(Pg*)*,* p=0.027 for PPD and p=0.001 for BOP.

b Interaction between baseline nadir CD4+ and log_10_(23s rDNA), p=0.031.

c Interaction between baseline nadir CD4+ and log_10_ *(Tf*)*,* p=0.006.

Exposure*: Nadir CD4+ T cell count is per 100 cells/µl increase; pathogens (*Pg,* 23s rDNA, *Td* and *Tf*) are per 1-log_10_ increase at baseline.

#### Longitudinal effects of immunological or virological markers on clinical measures of PD

There was no evidence of an independent association between longitudinal changes in CD4+ T-cell count or level of HIV RNA and longitudinal changes in any of the clinical measures of PD (see Table 4). 

### Discussion

In this observational study of HIV-infected adults on HAART experiencing immune reconstitution, we established that nadir CD4+ T-cell count influences periodontal disease in HIV-infected adults both prior to and after HAART initiation. Prior to HAART, HIV-related immunosuppression (as measured by nadir CD4+ T-cell count and baseline CD4+ T-cell count) was associated with worse periodontal disease, as measured by REC and CAL. We also found that during immune reconstitution on HAART, a lower nadir CD4+ T-cell count was associated with a greater longitudinal improvement in PDD, REC, and BOP in subjects with higher baseline quantities of periodontal pathogens as compared to subjects with lower baseline quantities of periodontal pathogens. Our results suggest that exposure to HIV-related immunosuppression worsens periodontal tissue loss; whereas, during immune reconstitution on HAART, an undefined mechanism may be contributing to improvement in periodontal disease. It should also be noted that PD improvement may in part be due to dental care received outside of the study and/or enhanced oral hygiene resulting from study-delivered oral health messages.

Because our subjects had only minimal HAART exposure at study enrollment (median 2.7 months) our baseline results should and do agree with prior pre-HAART era studies such as Robinson et al, 1996, McKaig et al, 1998 as well as HAART era cohorts with few subjects on HAART at baseline, such as Alves et al, 2006. All of these large-scale studies found significant cross-sectional associations between lower CD4+ T-cell count and REC and/or CAL[6,16,17]. Our cross-sectional findings differ from those of Drinkard et al, 1991, Friedman et al, 1991 and Scheutz et al, 1997, all of whom used partial mouth periodontal probing methodology [18,19,20] which is known to underestimate the prevalence of PD[21,22,23]. It is interesting to note that in the Women’s interagency HIV Study (WIHS), there were significant cross-sectional associations between HIV and loss of interdental papillae (i.e. loss of periodontal tissue); however, these findings were not interpreted as periodontal disease and therefore, no association between HIV and periodontal disease was reported[17,24]. For example, Mulligan et al, 2004, (the baseline WIHS study) found that HIV seropositive women had a significantly greater amount of abnormal (i.e., loss of) gingival papilla (p=0.004) as compared to an HIV-negative group of women[24]. Similarly, in the WIHS longitudinal report by Alves et al, 2006, HIV seropositve women had significantly greater clinical attachment loss at baseline than the HIV-negative comparison group (1.6 versus 1.1 mm, p=0.003); however, these authors focused on the lack of a longitudinal association between CD4+ T-cell count, HIV RNA and measures of periodontal disease progression, and did not clearly delineate the pre-HAART effects (i.e. at baseline) from the post-HAART effects (i.e., at the end of the longitudinal study) of HIV on PD[17]. 

To our knowledge, there has only been one other longitudinal epidemiological study examining the relationship between HIV and PD using traditionally-defined periodontal disease measures[17]. Alves et al, 2006, found that traditional markers of immune function (CD4+ T-cell count and HIV RNA) were not associated with longitudinal change in clinically measured periodontal disease. In our study, we found that the effect of nadir CD4+ T-cell count on periodontal disease during immune reconstitution was modified by the level of periodontal pathogens. Overall, our findings may suggest that, during immunosuppression, a dysregulated host immune response contributes to periodontal tissue loss in HIV infected adults; whereas, during immune reconstitution on HAART, host-mediated immunity re-establishes a more functional and effective response to periodontal pathogens in a dose-dependent manner. 

The importance of nadir CD4+ T-cell count has been well recognized in studies examining the link between HIV infection and cardiovascular disease risk[25,26,27]. Our findings herein, coupled with our 2009 report[5], highlight the importance of including nadir CD4+ T-cell count in future studies examining PD in the post-HAART era. Outside of our reports [5,8], nadir CD4+ T-cell count has been largely ignored in the dental literature; including this critical value may contribute to understanding the wide variability in reported levels of PD in HIV infected cohorts. Likewise, since the level of specific subgingival periodontal pathogens can influence host-mediated inflammation during immune reconstitution on HAART, measuring and analyzing specific periodontal pathogens may be an important variable in understanding the relationship between HIV and periodontal disease.

The unexpected finding of gingival recession appearing to improve during the study could be explained by a longitudinal increase in gingival volume and/or height (in a coronal direction) relative to the cemetoenamel junction. Such an effect may represent a local immune reconstitution inflammatory syndrome (IRIS)-like response. 

This study had a number of strengths. Our cohort was well characterized immunologically and subjects in our cohort had all recently initiated HAART. All subjects were taking HAART at baseline and at 97% of the study’s longitudinal follow-up visits. Periodontal disease was measured thoroughly and each clinical component (i.e., PPD, REC, CAL and BOP) was quantified as a continuous variable, as per expert guidelines[11,12]. Our findings further support the premise by Beck et al, 2002 that when examining periodontal disease (in this study as an outcome measure), the individual components of PD (i.e. PPD, REC and CAL) [11] may be differentially influenced by specific systemic exposures, namely, markers of host immune function. We also included both clinical and microbiological measures of PD. Like Lekakis et al, 2008, and others in the medical literature[27,28,29], we reported all critical immunological variables (See Table 1: i.e., nadir CD4+ T-cell count, time since first seropositive, time on HAART, etc.); these variables help characterize or “frame” our cohort relative to other cohorts of HIV infected adults. Thus, future HAART-era reports on this and other oral health-related topics should, at minimum, include their cohort’s median nadir CD4+ T cell count. 

This study also has limitations. Our sample size was relatively small and so there may have been limited power to detect clinically significant associations between immunological markers and clinical PD. Additionally, the Type I error rate was inflated because we took a hypothesis-generating approach to the analyses and did not adjust for multiple tests arising from the large number of models that were fit to estimate the association between multiple measures of immunological function and multiple clinical measures of PD. The inclusion of a comparison group was also not feasible; instead, we thoroughly characterized our adult cohort with HIV-infection[8]. Our cross-sectional and longitudinal findings can be compared most appropriately to HIV-positive cohorts with similar characteristics (i.e., similar demographic make-up, similar median nadir CD4+ T-cell count and those recently starting HAART). 

It is reasonable to conclude that those with exposure to lower CD4+ T-cell counts (accrued prior to the initiation of HAART) will tend to have increased gingival recession and increased clinical attachment loss as compared to those whose nadir CD4+ T-cell count remains higher. It should be noted that the poor periodontal health and low median nadir CD4 + T cell count of our cohort may both reflect poor access to care. Further, while HAART and/or immune reconstitution may help ameliorate PD, other factors, such as smoking status[23], access to ongoing dental care[30,31,32], dental anxiety[33], psychosocial stress, concomitant medical conditions, oral health literacy[34], oral hygiene skills[35]—and, as per this study, nadir CD4+ T-cell count, may influence the long-term risk for ongoing chronic periodontal disease in this population. 

In this study, it is interesting to note that although clinical measures of PD improved longitudinally, microbiological measures of PD remained relatively unchanged across time (see Vernon et al, 2011; Table 2)[8]. Baseline CD4+ T cell count and level of HIV RNA were not related to baseline levels of periodontal pathogens (data not shown). These findings tend to suggest that an altered host response—either innate and/or adaptive immunity—influences the relationship between HIV and periodontal disease. Future studies should examine the mechanism(s) whereby HIV-infection contributes to increased risk for recession and clinical attachment loss, and the long-term longitudinal effect on HIV and/or HAART on PD in those with higher and lower nadir CD4+ T-cell counts. These effects may be linked to elevated cytokine production or the abnormal innate immune cell (i.e., neutrophils[36], dendritic cells, monocytes[37], etc.) function. Such markers of innate and/or adaptive immunity may link HIV to periodontal disease improvement during immune reconstitution; if so, a greater understanding of this mechanism could offer insights into HIV pathogenesis and/or periodontal disease. Regardless of these scientific issues, it should be noted that even with improvement during relatively short-term immune reconstitution, levels of periodontal disease in our cohort remained elevated (see Table 2); thus, efforts to prevent and treat periodontal disease in HIV infected adults remain an ongoing public health and research priority.

## Supporting Information

Table S1 **2 Patient characteristics during follow-up time.**
(DOCX)
