# Supplementary material for: Effect of Nadir CD4+ T Cell Count on Clinical Measures of Periodontal Disease in HIV+ Adults before and during Immune Reconstitution on HAART
Source: PLoS One. 2013 Oct 11;8(10):e76986. doi: 10.1371/journal.pone.0076986 (PMC3795634; doi:10.1371/journal.pone.0076986)
Supplement: Table S1 — 2 Patient characteristics during follow-up time. (DOCX) [file pone.0076986.s001.docx]

**Table S1.2 Patient characteristics during follow-up time**

| **Characteristics after baseline** | **Mean (SD)**  **Frequency (Proportion)** |
| --- | --- |
| Total study follow-up time (months) | 22.8(10.3) |
| Total study visits (including baseline visit) | 3.55(1.20) |
| Total number of teeth extracted | 1.0(2.3) |
| Total number of teeth extraction across all patients during the study | 40 |
| Patients with no teeth extracted during the study | 29 (72.5%) |
| Patients with <3 teeth extracted during the study | 36 (90%) |
| Total visits to AEGD | 49.2(50.1) |
| Total visits on HAART | 97.1(10.6) |
| Total visits after receiving scaling and root planning (gross debridement or scaling and root planning) | 11.3(29.1) |
| Total recall visits seen by dentist since last visit | 53.5(36.1) |

AEGD, Advanced Education in General Density at Case Western Reserve University School of Dental

Medicine (a Ryan White provider); HAART, highly active antiretroviral therapy.
